# A case report of recurrent acute pancreatitis associated with life threatening atypical hemolytic uremic syndrome

**DOI:** 10.1097/MD.0000000000019731

**Published:** 2020-05-29

**Authors:** Elizabeth M. Jean-Marie, Jonathan J. Cho, Jose G. Trevino

**Affiliations:** a Department of Surgery, University of Florida Health Sciences Center; b Department of Anatomy and Cell Biology, College of Medicine, University of Florida, Gainesville, FL, United States.

**Keywords:** acute pancreatitis, alcohol induced pancreatitis, atypical hemolytic uremic syndrome, eculizumab, hemolytic anemia, hemolytic uremic syndrome, thrombocytopenia

## Abstract

**Introduction:**

Hemolytic uremic syndrome (HUS) is a thrombotic microangiopathy defined by the sudden onset of hemolytic anemia, thrombocytopenia, and acute kidney injury (AKI). HUS is categorized as either typical, caused by Shiga toxin-producing *Escherichia coli* infection, or atypical HUS (aHUS), usually complement mediated or secondary to systemic disease. We describe a rare case of aHUS in an adult male patient with recurrent acute pancreatitis.

**Patient clinical findings:**

A 32-year-old Caucasian male presented to our institution for his third episode of alcohol-induced pancreatitis. He presented with abdominal pain, elevated lipase and pancreatic inflammation on computed tomography consistent with acute pancreatitis. While admitted, he developed sudden onset severe thrombocytopenia, AKI and hemolytic anemia.

**Diagnosis, therapeutic interventions, outcomes:**

Peripheral blood smear, haptoglobin and hemoglobin level confirmed microangiopathic hemolytic anemia. Worsening anemia, thrombocytopenia and AKI were consistent with the diagnosis of aHUS. The patient's pancreatitis resolved with supportive measures, but resolution of significant thrombocytopenia and AKI was not achieved until administration of eculizumab, a complement inhibiting therapy. Eculizumab therapy provided dramatic improvement in this patient, with platelet count increasing from a low of 11,000 to >100,000 within 48 hours of therapy. Creatinine and hemoglobin levels returned to baseline within 3 weeks.

**Conclusion:**

Recurrent pancreatitis is suggested as the etiology of atypical HUS in this patient and this condition should be recognized and treated in a timely manner for optimal clinical outcomes.

## 1 Introduction

Hemolytic uremic syndrome (HUS) is a rare disease defined by the sudden onset of hemolytic anemia, thrombocytopenia, and acute kidney injury (AKI). With HUS, anemia is severe and microangiopathic in nature, presenting with schistocytes in the peripheral smear, high serum lactate dehydrogenase, and decreased serum haptoglobin level. Thrombocytopenia is typically severe as well, with a platelet count of <60,000/mm^3^ in most cases.^[1–3]^

The disease is most commonly triggered by Shiga-like toxin producing Escherichia coli, presenting with bloody diarrhea in children. Non-Shiga toxin-associated HUS, or atypical HUS (aHUS), is extremely rare, seen in adults with an incidence of 1 to 7 per 1,000,000 in the U.S. and Europe.^[4]^ Clinical outcomes for aHUS are poor, with up to 50% of cases progressing to end-stage renal failure and a 25% mortality rate during the acute phase if left untreated.^[4]^

Identified causes include complement disorders, drugs, cobalamin deficiencies and systemic diseases such as systemic lupus erythematous and severe pre-eclampsia.^[2]^ For complement mediated aHUS, studies have shown excellent clinical and laboratory response to Eculizumab, a C5 complement inhibitor that prevents formation of membrane attack complex.^[5]^

Atypical HUS secondary to acute pancreatitis has previously been reported in only a few cases in the literature.^[6–12]^ These cases have predominantly been treated with plasma exchange, apart from 1 reported successful treatment with eculizumab.^[13]^ Recurrent pancreatitis as a potential etiology for aHUS has scarcely been described, with only 1 previous case report.^[14]^ Here we provide a unique case of recurrent alcohol-induced pancreatitis complicated by aHUS that was successfully treated with eculizumab.

## 2 Case report

A 32-year-old Caucasian man with a past medical history of alcohol use disorder and recurrent pancreatitis presents with epigastric pain, nausea and vomiting for 2 days. This admission was this patient's third episode of pancreatitis. In his first hospitalization, pancreatitis (lipase 111 U/L) was complicated by pseudocyst formation and normocytic anemia (Hemoglobin 9.4-12.6 g/dL). Creatinine (0.83-1.05 mg/dL) and platelet count (456-560 × 10^3^/mm^3^) were within normal limits. He was managed with supportive care including maintenance intravenous fluids, analgesics, and direct jejunal enteral nutrition. Workup for etiology of pancreatitis included a lipid panel negative for hypertriglyceridemia (triglyceride 127 mg/dL), and a right upper quadrant ultrasound negative for sludge, cholelithiasis, or choledocholithiasis. Etiology of pancreatitis was attributed to alcohol use disorder with no role for gallbladder etiology, as the patient elected for cholecystectomy on an outpatient basis.

The patient presented again 7 months later and was admitted for his second episode of alcohol-induced pancreatitis. His second hospitalization was complicated by AKI (creatinine 1.6-12 mg/dL), thrombocytopenia (nadir 22 × 10^3^/mm^3^, peak 494 × 10^3^/mm^3^) and normocytic anemia (hemoglobin 7.7-16.6 g/dL). Workup for thrombotic thrombocytopenic purpura (TTP), HUS, heparin-induced thrombocytopenia was grossly negative on this admission, including ADAMTS13 activity (43%), C3 level (154 mg/dL), C4 level (41 mg/dL), negative heparin platelet antibody and a peripheral blood smear negative for schistocytes. Thrombocytopenia, and AKI resolved with supportive measurement and were attributed to a systemic inflammatory response syndrome during the second hospitalization.

On his third admission, he was admitted for management of acute pancreatitis evidenced by a lipase level of 1,019 U/L and computed tomography scan consistent with acute pancreatic inflammation. The etiology of acute pancreatitis was inferred to be alcohol since his phosphatidylethanol level was 464 ng/mL.

His vital signs were unremarkable and physical exam was significant for tenderness to palpation and voluntary guarding in the epigastric region. His basic metabolic profile and complete blood count were unremarkable. His hepatic function panel was significant for elevated total bilirubin of 2.4 mg/dL. He was started on maintenance intravenous fluids and had a nasojejunal tube placed for post-pyloric tube feeds.

On hospital day 2 he developed severe thrombocytopenia, with his platelet count decreasing to 48,000/ mm^3^. He also developed AKI, with an increase in creatinine from 1.37 mg/dL to 2.00 mg/dL. Workup for thrombocytopenia was significant for an elevated serum lactate dehydrogenase 1007 IU/L, and decreased serum haptoglobin 30 mg/dL. ADAMTS13 activity (59%), and coagulation studies were tested (Partial thromboplastin time 25-27 seconds; Prothrombin time 11.2-14.2 seconds; International Normalized Ratio 1.0-1.3). Total complement CH50 was elevated at 195 complement activity enzyme units (reference 60-144), and complement activity was elevated at 211 complement activity enzyme units (reference 60-144). Peripheral blood smear was negative for schistocytes at this time. C3 and C4 complement levels were within normal limits.

**Figure 1:**
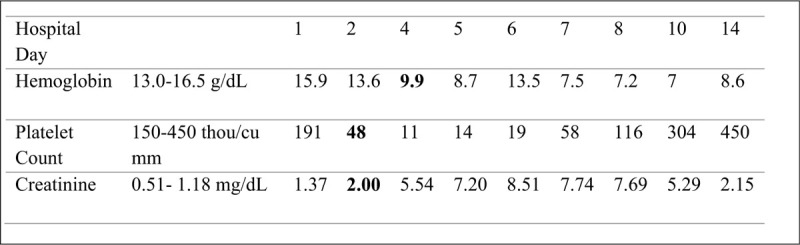
Establishing diagnosis of hemolytic uremic syndrome. Thrombocytopenia noted on hospital day 2 with platelet count of 48,000/cu mm. Acute kidney injury noted on hospital day 2 with creatinine rise to 2.00 mg/dL. Anemia noted on hospital day 4 with hemoglobin drop to 9.9 g/dL.

**Figure 2:**
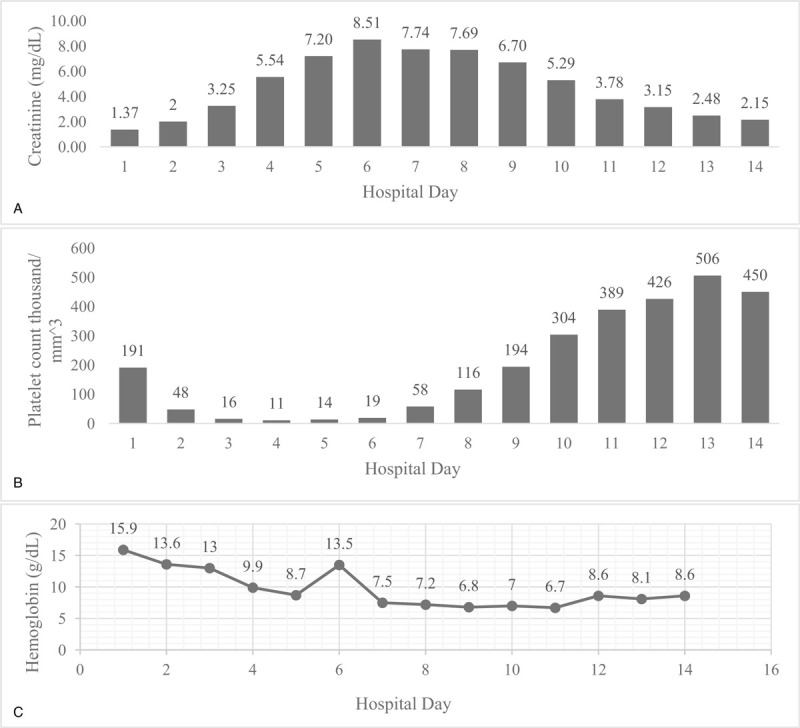
Thrombocytopenia, acute kidney injury and anemia along the 14 d hospital course. (A) Lowest platelet count noted on hospital day 4, thrombocytopenia begins to resolve after initiation of eculizumab treatment on hospital day 6. (B) Creatinine down trends after eculizumab treatment. (C) Anemia persisted throughout the hospital stay. One d of improvement noted on hospital day 6.

On hospital day 4, he developed anemia with a hemoglobin level of 9.9 g/dL. Thrombocytopenia continued to worsen, with a platelet count of 11,000/ mm^3^ and AKI continued to worsen as well, with a creatinine rising to 5.54 mg/dL. The patient did not have any evidence of bleeding, so no platelet transfusion was required. Peripheral blood smear was repeated the next day, showing burr cells and 4 schistocytes/high power field. Due to for concern for rapidly developing aHUS (Fig. 1), eculizumab 900 mg was administered on hospital day 6. Improvements on eculizumab were immediate and dramatic. Thrombocytopenia resolved within 3 days (Figs.1 and 2). Creatinine continued to trend down from a high of 8.51 mg/dL to 2.15 mg/dL on the day of discharge (Figs. 1 and 2). Creatinine returned to baseline 2 weeks after discharge (Figs. 1 and 2). Anemia did not resolve in the acute phase; patient required 2 red blood cell transfusions during this admission (Figs. 1 and 2).

Eculizumab 900 mg was given for 2 doses inpatient and continued weekly for 3 weeks outpatient for a total of 5 doses. Atypical HUS 12-gene panel testing of blood (Machaon Diagnostics Laboratory) revealed a heterozygous missense variant (c.389A>G, p. Tyr130Cys) in exon 3 of the *MMACHC* gene. At 2 months following completion of eculizumab therapy, he continued to maintain a normal platelet count (204,000 /mm^3) and showed no evidence of long term renal damage with creatinine returning to baseline (1.16 mg/dL).

## 3 Discussion

Atypical HUS or TTP is rarely associated with pancreatitis.^[6–12]^ TTP or aHUS as a complication of recurrent pancreatitis with subsequent plasma exchange treatment was presented in 1 case report.^[14]^ Clinical presentation and diagnosis of pancreatitis from various etiologies include alcohol use disorder, endoscopic retrograde cholangiopancreatography, biliary disease, and idiopathic, preceded these cases of aHUS or TTP,^[6–12]^ similar to the current case report. The complement system, an arm of the innate immune system, has been implicated in the pathogenesis of pancreatitis; however, the exact mechanisms underlying complement activation and the role of complement factors in pancreatitis has yet to be elucidated.^[15]^ Autoimmune pancreatitis has been shown to exhibit high levels of serum activated immune complex linked to the classic pathway.^[16]^ From the clinical presentation of the current case report of pancreatitis preceding atypical HUS, it may be plausible that acute recurrent pancreatitis may induce some degree of complement system dysfunction to engender atypical HUS.

Eculizumab was shown to inhibit complement-mediated thrombotic microangiopathy and induced time-dependent improvement in renal function in patients with atypical hemolytic-uremic syndrome.^[5]^ Eculizumab was only reported to successfully treat 1 reported case of endoscopic retrograde cholangiopancreatography-induced pancreatitis associated aHUS; however the patient in this report also received plasma exchange therapy.^[8]^ The resolution of AKI and thrombocytopenia in the setting of aHUS or TTP associated with acute or recurrent/chronic pancreatitis by eculizumab monotherapy has not been reported. In the current case report, the patient's AKI and thrombocytopenia started to resolve following the initiation of eculizumab monotherapy.

In the current case report, there is no strong evidence for complement mediated HUS due to negative aHUS 12-gene panel and normal C3 and C4 levels. However, a heterozygous missense variant (c.389A > G, p.Tyr130Cys) in exon 3 of the MMACHC gene was detected. This MMACHC gene variant was detected in a case report of 20 year-old male adult-onset Eculizumab-resistant HUS associated with cobalamin C deficiency as part of a compound heterozygosity of 2 known cobalamin C deficiency-causative mutations (c.271dupA and c.389A>G).^[17–19]^ It is highly improbable that a single missense variant c.389A > G of MMACHC gene caused aHUS in the current case report due to the heterozygosity and absence of another compound missense mutation in MMACHC. The rapid resolution of aHUS components of AKI and thrombocytopenia with eculizumab also cannot be explained by this MMACHC single missense variant c.389A > G.

Atypical HUS is rarely diagnosed in the adult population. Pancreatitis preceding aHUS clinical presentation of hemolytic anemia, thrombocytopenia, and AKI is also rare with only a limited number of case reports. Eculizumab was shown to be an effective treatment for atypical HUS in clinical trials. Elevating creatinine levels, signifying AKI, thrombocytopenia, and anemia in the setting of pancreatitis should raise the suspicion of aHUS, requiring further diagnostic workup such as peripheral blood smear and ADAMTS13 levels. Eculizumab may be considered to treat aHUS associated with pancreatitis in conjunction with supportive therapy for the pancreatitis. Future randomized clinical trials are needed to establish the therapeutic efficacy of eculizumab in non-complement mediated aHUS associated with pancreatitis.

## Acknowledgments

The authors thank the University of Florida, College of Medicine, Department of Surgery, Pancreatic, Liver, and Biliary Surgery service, and Department of Medicine, Hematology and Oncology Division for assistance in patient care.

## Author contributions

EMJM, JJC: medical students; JGT attending physician on the management team of the patient. EMJM, JJC, and JGT wrote the manuscript.

**Conceptualization:** Elizabeth Jean-Marie, Jonathan J. Cho, Jose G. Trevino.

**Data curation:** Elizabeth Jean-Marie, Jonathan J. Cho, Jose G. Trevino.

**Funding acquisition:** Jose G. Trevino.

**Investigation:** Elizabeth Jean-Marie, Jonathan J. Cho, Jose G. Trevino.

**Methodology:** Elizabeth Jean-Marie, Jonathan J. Cho, Jose G. Trevino.

**Project administration:** Jose G. Trevino.

**Supervision:** Jose G. Trevino.

**Validation:** Jonathan J. Cho, Jose G. Trevino.

**Writing – original draft:** Elizabeth Jean-Marie, Jonathan J. Cho, Jose G. Trevino.

**Writing – review & editing:** Elizabeth Jean-Marie, Jonathan J. Cho, Jose G. Trevino.
